# Effect of Immune Activation during Early Gestation or Late Gestation on Inhibitory Markers in Adult Male Rats

**DOI:** 10.1038/s41598-020-58449-x

**Published:** 2020-02-06

**Authors:** Tasnim Rahman, Cynthia Shannon Weickert, Lauren Harms, Crystal Meehan, Ulrich Schall, Juanita Todd, Deborah M. Hodgson, Patricia T. Michie, Tertia Purves-Tyson

**Affiliations:** 1 School of Psychiatry, Faculty of Medicine, University of New South Wales, Sydney, NSW Australia; 2 Neuroscience Research Australia, Sydney, NSW Australia; 3 Department of Neuroscience and Physiology, Upstate Medical University, Syracuse, NY USA; 4 School of Psychology, The University of Newcastle, Sydney, NSW Australia; 5 Priority Centre for Brain and Mental Health Research, The University of Newcastle, Newcastle, NSW Australia; 6 Hunter Medical Research Institute, Newcastle, NSW Australia; 7 Division of Psychology, School of Medicine, College of Health and Medicine, University of Tasmania, Hobart, TAS Australia; 8 School of Medicine and Public Health, The University of Newcastle, Newcastle, NSW Australia

**Keywords:** Disease model, Neuroscience, Schizophrenia

## Abstract

People with schizophrenia exhibit deficits in inhibitory neurons and cognition. The timing of maternal immune activation (MIA) may present distinct schizophrenia-like phenotypes in progeny. We investigated whether early gestation [gestational day (GD) 10] or late gestation (GD19) MIA, via viral mimetic polyI:C, produces deficits in inhibitory neuron indices (GAD1, PVALB, SST, SSTR2 mRNAs) within cortical, striatal, and hippocampal subregions of male adult rat offspring. *In situ* hybridisation revealed that polyI:C offspring had: (1) SST mRNA reductions in the cingulate cortex and nucleus accumbens shell, regardless of MIA timing; (2) SSTR2 mRNA reductions in the cortex and striatum of GD19, but not GD10, MIA; (3) no alterations in cortical or striatal GAD1 mRNA of polyI:C offspring, but an expected reduction of PVALB mRNA in the infralimbic cortex, and; (4) no alterations in inhibitory markers in hippocampus. Maternal IL-6 response negatively correlated with adult offspring SST mRNA in cortex and striatum, but not hippocampus. These results show lasting inhibitory-related deficits in cortex and striatum in adult offspring from MIA. SST downregulation in specific cortical and striatal subregions, with additional deficits in somatostatin-related signalling through SSTR2, may contribute to some of the adult behavioural changes resulting from MIA and its timing.

## Introduction

Neural activity between the cortex and striatum regulates motor, cognitive, and limbic function, and aspects of these modalities are perturbed in people with schizophrenia^1,2^. This neural activity is influenced by local inhibitory neurons, and the dysregulation of inhibition is postulated as a central contributor in the development of schizophrenia. Inhibitory tone is mainly established during early neurodevelopment to coordinate efficient neurotransmission^3,4^. Inhibitory interneurons migrate from the ganglionic eminence and the subventricular zone through the white matter to reach their destination in the cortical grey matter (see^5^ for review) and hippocampus (see^6^ for review). Striatal neurons are largely generated from the ganglionic eminences^7^. Perturbations during this neurodevelopmental epoch is therefore postulated to dysregulate inhibitory tone in the brain^8^ and contribute to the behavioural phenotypes in schizophrenia (see^9,10^ for review).

Glutamate decarboxylase-1 (GAD1) is one of the enzymes that catalyses the main inhibitory neurotransmitter in the brain, gamma-aminobutyric acid (GABA)^11^. There are two main subsets of GABAergic neurons that are distinguished based on their expression of the calcium-binding protein, parvalbumin (PVALB), or the neuropeptide hormone, somatostatin (SST) (see^12^ for review). Studies in post-mortem human brain show that schizophrenia cases have reduced cortical^13^, hippocampal^14,15^, and striatal^16,17^ GABAergic indices that suggest that these regions may contribute to the cognitive deficits exhibited by people with schizophrenia. The most consistently reported inhibitory deficits in the cortex of people with schizophrenia include reductions in *GAD1*^18,19^, *PVALB*^8,20^, and *SST*^8,21–24^ mRNAs. SST also elicits inhibition via somatostatin receptors (see^25^ for review), and decreases in SST receptor 2 (*SSTR2*) mRNA is reported in the dorsolateral prefrontal cortex (dlPFC) of people with schizophrenia^26^. Whether it is proliferation, differentiation, migration, survival and/or function of these inhibitory neurons that contribute to schizophrenia pathophysiology is unclear.

One way to test the gap in knowledge regarding the origin of inhibitory neuropathology involves recapitulating epidemiological factors that increase the risk of schizophrenia in animal models. Epidemiological investigations show gestational and perinatal infection increases the risk of schizophrenia in progeny^27–32^. In rodent studies, maternal immune activation (MIA) can be induced in pregnant dams with the viral mimic polyriboinosinic:polyribocytidilic acid (polyI:C). This results in offspring (henceforth referred to as polyI:C offspring) that exhibit schizophrenia-like neuroanatomical changes at adulthood (such as reduced brain volume in cortical, striatal, and hippocampal regions^33^) and schizophrenia-like behavioural changes (such as deficits in sensorimotor gating^34,35^, increased amphetamine-induced hyperlocomotion^34^, and impaired learning^36^). This suggests MIA may contribute to neurobiological alterations in these brain regions in offspring. Indeed, reductions in GABA in cortex^37^, and *GAD1* mRNA in cortex and hippocampus^38,39^ are found in polyI:C offspring. SST and PVALB neuron populations are trophically dependent on trkB^40–43^, and preliminary studies show alterations in the gene expression of trkB in the striatum of adult male polyI:C offspring^44^. This suggests that inhibitory neuron deficits may occur in the striatum of male polyI:C offspring.

The timing of MIA exposure is associated with distinct behavioural changes that may be associated with distinct neurobiological changes that contribute to these behaviours in offspring at adulthood^45–47^. Indeed, the timing of MIA exposure determines the extent and pattern of brain changes in foetal neurodevelopment (see^48^ for review). In mice, early gestation MIA exposure results in offspring with behaviours that mimic positive symptoms of schizophrenia, and late gestation MIA exposure results in offspring with more negative and cognitive schizophrenia-like symptoms^49^. In rats, we found that early gestation MIA exposure results in male offspring with sensorimotor gating deficits, whilst late gestation MIA exposure results in male and female offspring with both sensorimotor gating and working memory deficits^50^. This suggests that the timing of MIA exposure on related neurobiological changes may be more apparent in male offspring versus female offspring.

Based on key neurotransmitter systems implicated in schizophrenia, our previous studies on the timing of MIA probed dopaminergic^50^ and glutamatergic^51^ indices in this model. For example, we recently reported that polyI:C offspring, from either early gestation MIA or late gestation MIA, exhibit glutamatergic alterations that are more pronounced in male offspring, and thus may relate to the sensorimotor gating deficits that are exacerbated in male polyI:C offspring^51^. Although studies in mice show that MIA is sufficient to reduce PVALB-positive cells in the medial prefrontal cortex in both early gestation and late gestation adult^49,52^ and juvenile^53^ polyI:C offspring, to our knowledge, there have been no studies on the effects of MIA, or the timing of MIA, on inhibitory neuron markers in the cortex, striatum, and hippocampus in rats.

The initial formation of the ganglionic eminence occurs at gestational day (GD) 10 ^54^, whereas interneurons (from the subventricular zone into the cortical plate) tangentially migrate from GD18 into the first postnatal weeks^55,56^. As the development of cortical inhibitory interneurons is a relatively protracted process, we hypothesised that the impact of gestational inflammation on inhibitory markers in different brain regions at adulthood would vary depending on the timing of MIA. Therefore, in the current study we investigated the effects of early gestation (GD10) or late gestation (GD19) polyI:C-induced MIA on gene expression of inhibitory indices (*GAD1*, *PVALB*, *SST*, and *SSTR2*) in the cortex, striatum, and hippocampus of adult male offspring. We hypothesised that late gestation polyI:C offspring would exhibit greater reductions in inhibitory mRNAs versus early gestation polyI:C offspring. Additionally, from previous investigations in polyI:C mouse offspring^49^, we hypothesised that polyI:C male offspring (GD10 and GD19 combined) have reduced *PVALB* mRNA in infralimbic cortex *a priori*. Finally, since inhibitory neurons transverse larger distances to reach the frontal cortex and hippocampus in comparison to the striatum, we hypothesised that polyI:C male offspring would exhibit more profound alterations in the cortex and hippocampus versus the striatum.

## Methods

### Animals and prenatal polyI:C administration

Experiments were performed in accordance with the National Health and Medical Research Council’s *Australian code for the care and use of animals for scientific purposes (*https://www.nhmrc.gov.au/guidelines-publications/ea28). The current study was approved by the University of Newcastle’s Animal Care and Ethics Committee (Approval number A-2009-108). Rats were sourced from the University of Newcastle’s Central Animal House and housed in conventional open-top cages with ad libitum food (standard rat chow, Specialty Feeds, WA, Australia) and water and 12 h light exposure in the University of Newcastle’s Behavioral Sciences Animal Facility.

Nulliparous postnatal day (PND) 70–90 Wistar rats were time-mated and day of vaginal plug detection was designated as GD0. Pregnant rats were randomly assigned one of two timing groups: GD10 (*n* = 8) or GD19 (*n* = 7). On the appropriate GD, pregnant rats were weighed, lightly anesthetized with isoflurane, and injected intravenously through the tail vein with 0.1 M phosphate-buffered saline (PBS) (control; *n* = 8) or 4 mg/mL of polyI:C (P9582, Sigma-Aldrich; *n* = 7) in PBS at a volume of 1 mL/kg body weight. To confirm successful MIA, saphenous vein blood samples were collected 2 hours after treatment injections. Plasma was used for interleukin-6 (IL-6) measurement using rat IL-6 Quantikine ELISA (R&D Systems, MN, USA). PolyI:C treated dams had significantly increased IL-6 levels (624.7 ± 57.0 pg/mL) compared to saline-injected dams (68.4 ± 57.0 pg/mL) (*F*_(1,8)_ = 50.56, *p* < 0.001). There was no effect of gestational timing (F_(1,8)_ = 0.24, *p* > 0.05) or interaction between gestational timing and treatment (*F*_(1,8)_ = 0.23, *p* > 0.05) on maternal plasma IL-6 levels.

Offspring were weaned on PND 21, separated into same-sex cages in pairs. Male adult offspring from either treatment or timing group did not differ in weight at time of euthanasia (GD10 control: 432 ± 32 g, *n* = 8; GD19 control: 441 ± 44 g, *n* = 8; GD10 MIA: 418 ± 47 g, *n* = 6; GD19 MIA: 415 ± 28 g, *n* = 6; treatment: *F*_(1,24)_ = 1.74, timing: *F*_(1,24)_ = 0.032, treatment × timing: *F*_(1,24)_ = 0.18, all *p* > 0.05). No more than two male offspring per litter were used in each experimental group to avoid litter-effect confounds and were randomly assigned to the study. Whole brains, snap-frozen in isopentane (−40 °C) for storage (−80 °C), were sectioned (coronal, 14 μm) using a cryostat (Leica, Wetzlar, Germany) and mounted onto gelatin-coated glass slides.

### *In situ* hybridisation

Riboprobes (Supplementary Table 1) were generated with ^35^S-UTP (CAT# NEG039H001MC Perkin Elmer, Waltham, Massachusetts, USA) using an *in vitro* transcription kit (CAT# P1121, Promega, Madison, Wisconsin, USA). *In situ* hybridisation was performed as previously described^57^, using 5 ng/ml radiolabelled riboprobes in hybridisation buffer, and ^35^S-UTP labelled sense strand riboprobes as a negative control (Supplementary Figures 1 and 2). Slides were exposed to BioMax MR (Kodak, Rochester, NY, USA) autoradiographic film (Supplementary Table 1) alongside a ^14^C standard slide (American Radiolabelled Chemicals, St. Louis, MO, USA).

### Quantification of mRNAs

**Figure 1:**
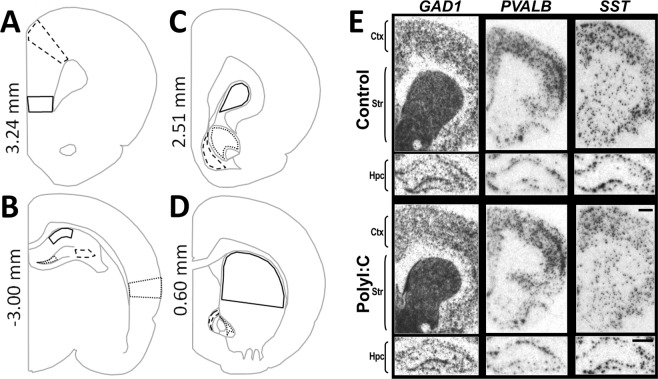
Gene expression of inhibitory-related markers quantified in cortex (Ctx), striatum (Str), and hippocampus (Hpc) subregions from representative *in situ* hybridization films when male offspring from vehicle (Control) or polyI:C (4 mg/kg) treated dams reached adulthood. (**A–D**) Grey line schematics of coronal sections of rat brain show the cortical (Ctx, **A,B**), striatal (Str, **C,D**), and hippocampal (Hpc, **B**) subregions (black outlines from −3.00 to 3.24 mm bregma) that were quantified in this study. (**A,B**) In rostral (**A**) and caudal (**B**) cortical sections, three subregions were quantified: infralimbic (solid line), cingulate (dashed line), and auditory (dotted line on the right-most side) cortex. (**B**) In rostral hippocampal sections, three subregions were quantified: CA1 (solid line), CA3 (dashed line), and CA4 (dotted line). (**C,D**) In rostral (**C**) and middle (**D**) striatal sections, three subregions were quantified: dorsal striatum (solid line), nucleus accumbens core (dotted line), and nucleus accumbens shell (dashed line). (**E**) Representative autoradiographs of glutamate decarboxylase-1 (*GAD1*) mRNA, parvalbumin (*PVALB*) mRNA, and somatostatin (*SST*) mRNA *in situ* hybridization films from control and polyI:C offspring. Sections pictured are from postnatal day 70–81 offspring from the GD10 group. The representative Ctx and Str autoradiographs are from ~2.00 mm bregma for *GAD1* mRNA, 2.50 mm for *PVALB* mRNA, and 1.70 mm bregma for *SST* mRNA. The representative Hpc autoradiographs are from ~−3.0 mm bregma. Statistics of treatment effects and treatment × subregion interactions are detailed in Table 1 and shown graphically in Fig. 4; there were no treatment × timing interactions for any of the markers. Bregma level (mm) is indicated on the left of the schematics. Scale bar = 1000 µm.

Developed films were digitised (600dpi, CAT# 8600 F, Canoscan, Canon Inc, Japan) and calibrated using NIH imaging software (v1.56; http://rsb.info.nih.gov/nih-image) to produce nCi/mg tissue equivalent (t.e.) values based on the standard Rodbard curve obtained from the ^14^C standards to remove background intensity. Optical density values were quantitated using ImageJ (v1.48; https://imagej.nih.gov/ij/) and averaged across hemispheres from two to four sections per animal. Cortical [infralimbic (IL), cingulate (Cg), auditory (Aud), Fig. 1A,B], striatal [dorsal striatum (DS), nucleus accumbens core (AC), nucleus accumbens shell (AS), Fig. 1C,D], and hippocampal [cornu ammonis area 1(CA1), cornu ammonis area 3 (CA3), cornu ammonis area 4 (CA4), Fig. 1B] regions were identified as per Paxinos 6^th^ edition Rat Atlas^58^.

### Statistical analysis

Graphs were plotted using Graph Pad Prism (v6) and data analysed with IBM SPSS statistics (v23). All data passed Shapiro-Wilk normality tests. Three-way mixed analysis of variance (ANOVA) was conducted for each region (cortex, striatum, and hippocampus), separately; hence there were three 2 × 2 × 3 repeated-measure (RM) ANOVAs in total. For each of these analyses, the two between-subject factors were treatment (polyI:C or vehicle) and timing (GD10 or GD19), and the within-subject factor was subregions (cortical subregions: IL, Cg, and Aud cortex; striatal subregions: DS, AC, and AS; hippocampal subregions: CA1, CA3, and CA4). Bonferroni tests were used for pairwise comparisons when the overall ANOVA was significant. The Greenhouse Geisser correction was used if Mauchley’s sphericity test was violated for within-subjects interaction effects. Deming regressions were conducted to query the relationship between each offsprings’ regional gene expression of each marker and their respective maternal IL-6 protein exposure. Data are expressed as the mean ± standard error of the mean (SEM) and two-sided *p* < 0.05 was deemed statistically significant.

## Results

**Figure 2:**
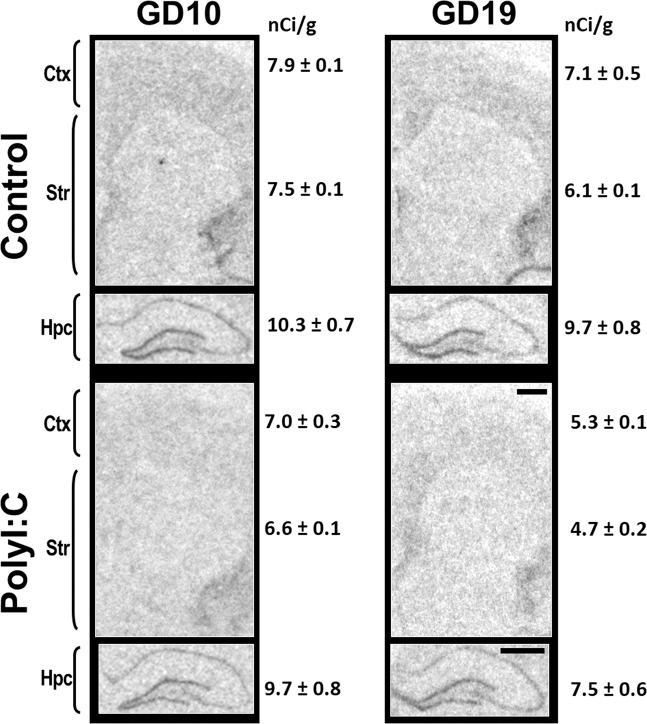
Representative autoradiographs of somatostatin receptor 2 (*SSTR2*) mRNA *in situ* hybridisation. Pregnant dams were treated with vehicle (Control, top) or 4 mg/kg polyriboinosinic:polyribocytidilic acid (PolyI:C, bottom) during early gestation (GD10, left) or late gestation (GD19, right). Coronal sections from ~2.3 mm bregma for cortex (Ctx) and striatum (Str), and ~−3.0 mm bregma for hippocampus (Hpc) were processed when offspring reached adulthood. GD19 polyI:C offspring had significant reductions of *SSTR2* mRNA in cortex and striatum compared to controls. Sections pictured are from postnatal day 77–84 offspring. Statistics of treatment × timing interactions for *SSTR2* mRNA are shown in Table 1 and shown graphically in Fig. 3. Scale bar = 1000 µm. The values to the right of the figures denote the average signal intensity ± S.E.M. (nCi/g) of the three cortical [infralimbic (not visible on image), cingulate, auditory (not visible on image)], striatal (dorsal striatum, nucleus accumbens shell, nucleus accumbens core), and hippocampal (CA1, CA3, DG) subregions quantified from the animals portrayed in the representative autoradiographs.

*GAD1* mRNA signals were punctate in the cerebral cortex and hippocampus, and darker and more homogeneous across the striatum (Fig. 1E). *PVALB* mRNA signals were punctate in the cerebral cortex, hippocampus, and striatum. *PVALB* mRNA signals in the cortex was stronger and denser in comparison to signals in the hippocampus and striatum (Fig. 1E). *SST* mRNA signals were punctate in all regions, and denser in the cerebral cortex in comparison to the hippocampus and striatum (Fig. 1E). *SSTR2* mRNA signals were highest in the molecular layer of the hippocampus, followed by the deeper layers of the cerebral cortex, the superficial layers of the cerebral cortex, and then the striatum (Fig. 2).

*GAD1* mRNA^59,60^, *PVALB* mRNA^61^, and *SST* mRNA^62^ signals correspond to previously described distributions. Although cortical *SSTR2* mRNA signals were lighter in comparison to some studies^63^, the distribution corresponds to *SSTR2* mRNA and SSTR2 binding results in multiple other studies (Supplementary Figure 1A)^64–68^. Control (sense) riboprobe hybridization, for all transcripts, was at background levels (Supplementary Figure 1B, 2).

**Figure 3:**
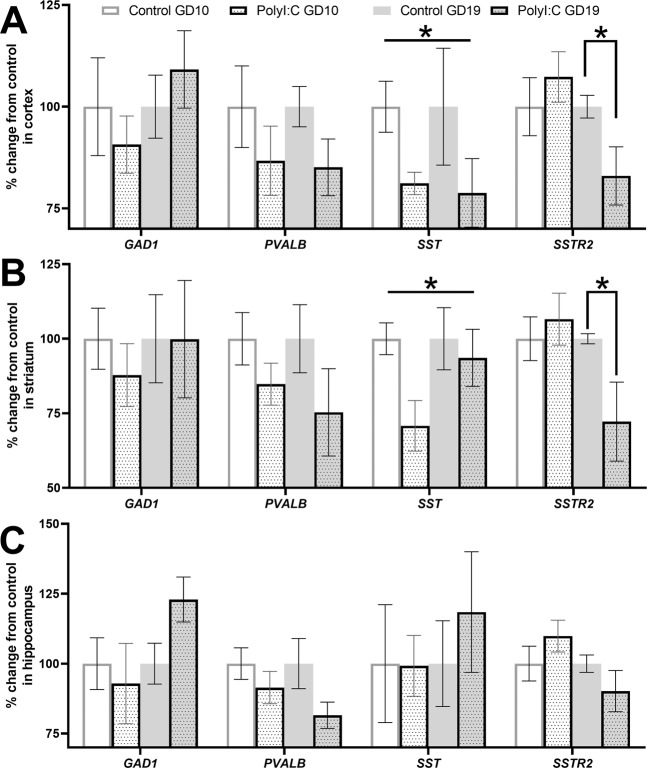
Somatostatin receptor 2 (*SSTR2*) mRNA is decreased in offspring exposed to polyI:C on GD19, not GD10, in cortex and striatum. Pregnant dams were treated with vehicle (control, solid bar) or 4 mg/kg polyI:C, (dotted bar) during early gestation (GD10, white) or late gestation (GD19, grey). Glutamate decarboxylase-1(*GAD1*), parvalbumin (*PVALB*), somatostatin (*SST*), and somatostatin receptor 2 (*SSTR2*) mRNAs were quantified in (**A**) cortex, (**B**) striatum, and (**C**) hippocampus from adult offspring. Data shown are pooled over three subregions for each region (cortex, striatum, and hippocampus. There was no overall treatment or treatment × timing interaction effect for *GAD1* or *PVALB* mRNAs in any region. There was an overall treatment effect for *SST* mRNA in (**A**) cortex and (**B**) striatum, but not (**C**) hippocampus, (refer also to Table 1). There was a treatment × timing interaction for *SSTR2* mRNA in (**A**) cortex and (**B**) striatum, but not (**C**) hippocampus, (refer also to Table 1). At GD10, there was no change in *SSTR2* mRNA levels observed between control and polyI:C offspring. At GD19, polyI:C offspring showed reduced *SSTR2* mRNA in (**A**) cortex by 29%, and in (**B**) striatum by 17% versus control offspring. Data are mean ± SEM (n = 6–8 rats per treatment and timing group). *p < 0.05.

**Figure 4:**
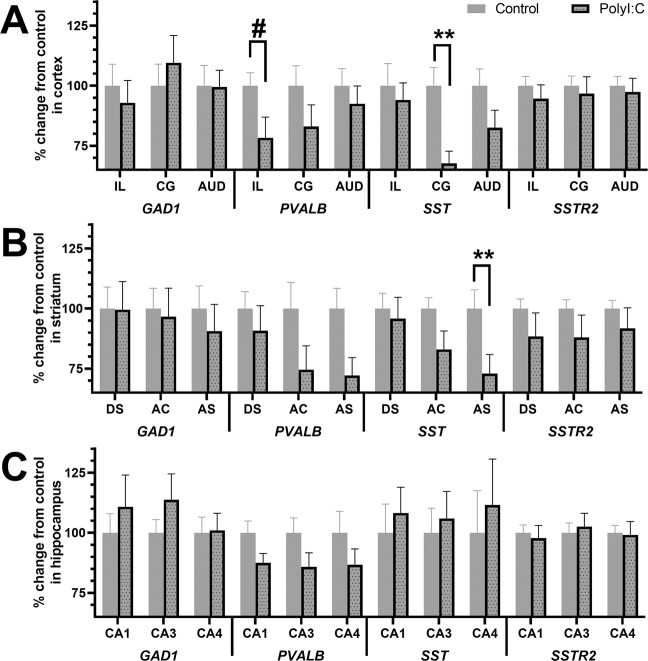
Inhibitory neuron gene expression is altered in subregions of (**A**) cortex and (**B**) striatum, but not (**C**) hippocampus, in adult male polyI:C offspring. Pregnant dams were treated with vehicle (Control, grey) or 4 mg/kg (PolyI:C, dotted) during either early gestation or late gestation (data herein represent gestational timing combined, data is presented separately in Supplementary Fig. 3). Glutamate decarboxylase 1 (*GAD1*) parvalbumin (*PVALB*), somatostatin (*SST*) and somatostatin receptor 2 (*SSTR2*) mRNAs were quantified in vehicle (control, grey bar) and polyI:C (dotted bar) offspring. Treatment × subregion interaction effects were not present for *GAD1* or *SSTR2* mRNAs in any subregion. PolyI:C offspring had reductions in *PVALB* mRNA in the infralimbic cortex compared to controls (*a priori* univariate ANOVA: F(1, 22) = 5.08, p = 0.04). *SST* mRNA was significantly reduced in (**A**) cingulate cortex and (**B**) nucleus accumbens shell of the striatum in polyI:C offspring. Refer to Table 1 for treatment × subregion interaction for *SST* mRNA. Data are mean ± SEM (n = 12–16 rats per group). Infralimbic cortex (IL), cingulate cortex (CG), auditory cortex (AUD), dorsal striatum (DS), nucleus accumbens core (AC), nucleus accumbens shell (AS), cornu ammonis (CA). ^#^*p* < 0.05, **p < 0.01.

There was a main effect of subregion on the expression pattern of *GAD1* mRNA, *PVALB* mRNA, *SST* mRNA, and *SSTR2* mRNA for cortical, striatal, and hippocampal comparisons in all offspring, except for *SST* mRNA in hippocampus (Supplementary Table 2). A summary of all treatment effects and treatment-related interactions are highlighted in Table 1. Briefly, there were significant treatment effects for *SST* mRNA in cortex (Fig. 3A) and striatum (Fig. 3B), treatment × timing interactions for *SSTR2* mRNA in cortex (Fig. 3A) and striatum (Fig. 3B), and treatment × subregion interactions for *SST* mRNA in cortex (Fig. 4A) and striatum (Fig. 4B). There were no significant treatment × timing × subregion interactions for any of the markers (Supplementary Figure 3A–C).

**Table 1:** Treatment effects and treatment interactions for inhibitory-related transcripts in cortex, striatum, and hippocampus. Two-way repeated measure ANOVAs [between-subject variables: treatment (control, polyI:C) and timing (GD10, GD19); within-subject variables: three subregions] were conducted per transcript per brain region. Significant values (*p* < 0.05) are bold and accompanied with an asterisk (*). Treatment effects for *SST* mRNA are shown in Figs. 1 and 2. Treatment × timing interactions for *SSTR2* mRNA are shown in Figs. 2 and 3. Treatment × subregion interactions for *SST* mRNA are shown in Fig. 1 and Supplementary Fig. 3. *p < 0.05.

Transcript	Brain region	Treatment		Treatment × Timing		Treatment × Subregion		Treatment × Timing × Subregion	
		*F*	*p*	*F*	*p*	*F*	*p*	*F*	*p*
*GAD1*	Cortex	0.001	0.97	0.912	0.349	0.403	0.61	1.709	0.2
	Striatum	0.099	0.755	0.092	0.764	0.601	0.469	0.247	0.66
	Hippocampus	0.56	0.461	2.213	0.15	0.846	0.416	0.428	0.614
*PVALB*	Cortex	2.868	0.106	0.012	0.914	0.241	0.741	0.843	0.418
	Striatum	3.286	0.084	0.127	0.725	1.15	0.321	0.29	0.721
	Hippocampus	3.716	0.066	0.484	0.494	0.879	0.415	3.176	0.056
*SST*	Cortex	4.772*	0.043*	0.078	0.783	3.973*	0.035*	0.089	0.89
	Striatum	4.926*	0.036*	2.33	0.14	5.532*	0.012*	1.072	0.338
	Hippocampus	0.279	0.602	0.324	0.575	0.129	0.825	2.248	0.132
*SSTR2*	Cortex	0.817	0.376	4.533*	0.045*	0.226	0.752	0.748	0.454
	Striatum	1.71	0.203	4.508*	0.044*	2.127	0.133	1.521	0.23
	Hippocampus	0.017	0.899	3.067	0.093	0.88	0.399	0.618	0.506

### Cortical *PVALB* mRNA and *SST* mRNA are reduced in polyI:C offspring in a subregion-specific manner, whereas cortical *SSTR2* mRNA is reduced in polyI:C offspring in a timing-specific manner

*GAD1* mRNA was not changed by MIA exposure in cortex (*F*_(1, 24)_ < 0.01, *p* = 0.97; Fig. 3A). Additionally, there were no treatment × timing (*F*_(1, 24)_ = 0.91, *p* = 0.35; Fig. 3A), treatment × subregion (Fig. 4A) or treatment × timing × subregion (*F*_(1.5,35.5)_ < 1.0, *p* > 0.05; Supplementary Figure 3A) interaction effects on *GAD1* mRNA (Table 1).

There was no overall effect of MIA treatment (*F*_(1, 20)_ = 2.87, *p* = 0.11; Fig. 3A) on *PVALB* mRNA in cortex. Additionally, there were no treatment × timing (*F*_(1, 20)_ = 0.01, *p* = 0.91; Fig. 3A), treatment × subregion (Fig. 4A) or treatment × timing × subregion (*F*_(1,40)_ < 1.0, *p* > 0.05; Supplementary Figure 3A) interaction effects on *PVALB* mRNA (Table 1). As expected, *PVALB* mRNA was reduced by 22% in the IL cortex of polyI:C offspring (*a priori* univariate ANOVA: *F*_(1, 22)_ = 5.08, *p* = 0.04; Fig. 4A).

There was a significant effect of MIA treatment on *SST* mRNA cortex, where polyI:C offspring had 20% less *SST* mRNA versus controls (*F*_(1,17)_ = 4.77, *p* = 0.04; Fig. 3A). There were no treatment × timing (*F*_(1, 17)_ = 0.08, *p* = 0.78; Fig. 3A) or treatment × timing × subregion (*F*_(2,34)_ = 0.09, *p* = 0.92; Supplementary Figure 3A) interaction effects on *SST* mRNA (Table 1). There was a treatment × subregion interaction effect (*F*_(2,34)_ = 3.97, *p* = 0.03, Fig. 4A) where polyI:C offspring had 33% less *SST* mRNA in Cg cortex (*F*_(1,17)_ = 9.03, *p* < 0.01), but not in IL cortex (*F*_(1,17)_ = 0.19, *p* = 0.67) or in Aud cortex (*F*_(1,17)_ = 2.78, *p* = 0.11), compared to control offspring.

There was no overall effect of MIA on *SSTR2* mRNA in cortex (*F*_(1, 22)_ = 0.82, *p* = 0.38; Fig. 3A). However, there was a significant treatment × timing interaction effect (*F*_(1, 22)_ = 4.53, *p* = 0.04) where MIA offspring had 14% less *SSTR2* mRNA in cortex at GD19 (*F*_(1,22)_ = 4.49, *p* < 0.05; Fig. 3A grey dotted bars), but not GD10 (*F*_(1,22)_ = 0.77, *p* = 0.39; Fig. 3A white dotted bars), compared to controls (Fig. 3A white and grey solid bars). There were no treatment × subregion (Fig. 4A) or treatment × timing × subregion (Supplementary Figure 3A) interaction effects on *SSTR2* mRNA (*F*_(2,44)_ < 1.0, *p* > 0.05, Table 1).

### Striatal *SST* mRNA is reduced in polyI:C offspring in a subregion-specific manner, whereas striatal *SSTR2* mRNA is reduced in polyI:C offspring in a timing-specific manner

*GAD1* mRNA (*F*_(1, 24)_ = 0.10, *p* = 0.76) and *PVALB* mRNA (*F*_(1, 22)_ = 3.29, *p* = 0.08) were not affected in the striatum by MIA (Fig. 3B). There were no treatment × timing effects for *GAD1* mRNA (*F*_(1, 24)_ = 0.09, *p* = 0.76) or *PVALB* mRNA (*F*_(1, 22)_ = 0.13, *p* = 0.73) in striatum (Fig. 3B). There were also no treatment × subregion (Fig. 4B) or treatment × timing × subregion interaction effects (Supplementary Figure 3B) for *GAD1* mRNA (*F*_(1.2,28.0)_ < 1.0, *p* > 0.05) or *PVALB* mRNA (*F*_(2,44)_ < 1.15, *p* > 0.05; Table 1).

There was a significant effect of MIA treatment on *SST* mRNA striatum, where polyI:C offspring had 17% less *SST* mRNA versus controls (*F*_(1,24)_ = 4.93, *p* = 0.04; Fig. 3B). There were no treatment × timing (*F*_(1, 24)_ = 2.33, *p* = 0.14; Fig. 3B) or treatment × timing × subregion (*F*_(1.6,37.6)_ = 1.07, *p* = 0.35; Supplementary Figure 3B) interaction effect on *SST* mRNA. There was a treatment × subregion interaction effect (*F*_(1.6,37.6)_ = 5.53, *p* < 0.01) where polyI:C offspring had 29% less *SST* mRNA in the nucleus accumbens shell (AS) (*F*_(1,24)_ = 7.37, *p* = 0.01; Fig. 4B), but not dorsal striatum (DS) (*F*_(1,24)_ = 0.366, *p* = 0.51; Fig. 4B), compared to control offspring. The pairwise comparison of *SST* mRNA in the nucleus accumbens core (AC) did not reach significance (*F*_(1,24)_ = 3.89, *p* = 0.06; Fig. 4B).

There was no overall effect of MIA on *SSTR2* mRNA in striatum (*F*_(1, 24)_ = 1.71, *p* = 0.20; Fig. 3B). However, there was a significant treatment × timing interaction effect (*F*_(1,24)_ = 4.51, *p* = 0.04) where MIA offspring had 33% less *SSTR2* mRNA in striatum at GD19 (*F*_(1, 24)_ = 5.89, *p* = 0.02; Fig. 3B), but not GD10 (*F*_(1,24)_ = 0.33, *p* = 0.57; Fig. 3B), compared to controls. There were no treatment × subregion (Fig. 4B) or treatment × timing × subregion (Supplementary Figure 3B) interaction effects on *SSTR2* mRNA (*F*_(2,48)_ < 2.13, *p* > 0.05, Table 1).

### Hippocampal gene expression of *GAD1*, *PVALB*, *SST*, or *SSTR2* is not altered in polyI:C offspring in a subregion- or timing-specific manner

MIA exposure did not significantly alter *GAD1* mRNA (*F*_(1, 24)_ = 0.56, *p* = 0.46), *PVALB* mRNA (*F*_(1, 23)_ = 3.72, *p* = 0.07), *SST* mRNA (*F*_(1,23)_ = 0.28, *p* = 0.60), or *SSTR2* mRNA (*F*_(1,24)_ = 0.24, *p* = 0.63) in hippocampus (Fig. 3C). Further, there were no treatment × timing interaction effects for *GAD1* mRNA (*F*_(1, 24)_ = 2.21, *p* = 0.15), *PVALB* mRNA (*F*_(1, 23)_ = 0.48, *p* = 0.49), *SST* mRNA (*F*_(1, 23)_ = 0.32, *p* = 0.58), or *SSTR2* mRNA (*F*_(1, 24)_ = 2.62, *p* = 0.12) in hippocampus (Fig. 3C). Finally, there were no treatment × subregion (Fig. 4C) or treatment × timing × subregion (Supplementary Figure 3C) interaction effects on *GAD1* mRNA (all *F*_(1.6,39.1)_ < 0.85, *p* > 0.05), *PVALB* mRNA (all *F*_(2,46)_ < 3.18, *p* > 0.05), *SST* mRNA (all *F*_(1.5,35.2)_ < 2.25, *p* > 0.05), or *SSTR2* mRNA (all *F*_(2,48)_ < 1.72, *p* > 0.05) in hippocampus (Table 1).

### Maternal IL-6 response to polyI:C treatment is related to *SST* mRNA in the cortex and striatum, but not hippocampus of offspring

Across all offspring (treatment and timings combined), there was a significant negative relationship between maternal IL-6 protein two hours after treatment and adult offspring *SST* mRNA in cortex (*F*_(1,18)_ = 4.39, *p* = 0.05) and striatum (*F*_(1,21)_ = 9.59, *p* < 0.01), but not hippocampus (*F*_(1,20)_ = 0.44, *p* = 0.52). The slopes were of moderate strength (cortex: R^2^ = 0.196, striatum: R^2^ = 0.314; Supplementary Figure 4). No significant relationships were found between maternal IL-6 protein two hours after treatment and adult offspring *GAD1* mRNA (cortex: *F*_(1,21)_ = 0.16, all *p* = 0.70; striatum: *F*_(1,21)_ = 0.19, all *p* = 0.67; hippocampus: *F*_(1,21)_ = 0.35, *p* = 0.56), *PVALB* mRNA (cortex: *F*_(1,17)_ = 0.64, *p* = 0.43; striatum: *F*_(1,19)_ = 2.58, all *p* = 0.12; hippocampus: *F*_(1,20)_ = 0.39, *p* = 0.54), or *SSTR2* mRNA (cortex: *F*_(1,21)_ = 0.46, *p* = 0.50; striatum: *F*_(1,21)_ = 1.74, all *p* = 0.20; hippocampus: *F*_(1,21)_ = 4.04, *p* = 0.06) levels in any brain region (Supplementary Figure 4).

## Discussion

Our findings demonstrate differential, long-term impacts of early gestation and late gestation MIA on inhibitory indices within the cortex and striatum of adult rat polyI:C offspring. As hypothesised, we found *PVALB* mRNA reductions in the infralimbic cortex of polyI:C offspring, which aligns with previous studies that report reductions in PVALB-positive cells in the medial prefrontal cortex in both early gestation and late gestation adult^49,52^ and juvenile^53^ mouse polyI:C offspring. Somewhat surprisingly, we did not find significant changes in the inhibitory neuron marker, *GAD1* mRNA, in cortical subregions, and no significant changes in either *PVALB* or *GAD1* mRNAs in the hippocampus of polyI:C offspring. Although this lack of apparent change in *GAD1* mRNA aligns with the majority of the reports in polyI:C offspring, methodological variations may contribute to inconsistent findings in the field (see Supplementary Table 3 for a brief overview). We also report no significant changes in either *PVALB* or *GAD1* mRNA in the striatum. Although, to our knowledge, there have been no investigations of *PVALB* gene expression in the striatum of adult polyI:C offspring, our results align with studies that also find no significant change in *GAD1* mRNA in the striatum of GD14 polyI:C adult rat offspring^38^. Our findings suggest that some inhibitory neuron deficits may not consistently result from MIA, or may arise due to MIA at a different gestational time point. Further, we acknowledge that our sample size may not detect subtle changes. Indeed, we find approximate trend reductions in *PVALB* mRNA in the cortex (*p* < 0.11), striatum (*p* < 0.09) and hippocampus (*p* < 0.07) of MIA offspring, and qualitative observation of our data suggests that this may arise from MIA at GD19. Our data suggests that future studies are warranted with larger sample sizes to investigate the effect of MIA timing on these inhibitory markers, particularly GAD1 and PVALB.

We present the first report of reduced *SST* mRNA in both cortex and striatum in the rat MIA model of schizophrenia. In the cortex of people with schizophrenia, *SST* mRNA reductions are typically of a larger magnitude and can be as robust (if not more robust) than the more often studied *PVALB* mRNA changes^8,20,21,24^, with widespread decreases in *SST* mRNA reported in dlPFC, orbital frontal cortex, anterior cingulate cortex, motor cortex, and visual cortex^8,20,21,24^. The anatomically specific MIA-induced reductions in *SST* mRNA in the cingulate cortex, but not in infralimbic and auditory cortex, suggest that MIA may impact specific circuitry differentially. If MIA alone is not sufficient to recapitulate the fairly pervasive cortical reductions in *SST* mRNA observed in people with schizophrenia^8,20,21,24,69^, a subsequent postnatal stressor, or ‘second hit’, may be needed to elicit a widespread change in *SST* gene expression in the MIA model [see^70^ for review]. The *SST* mRNA deficits detected in the cortex and striatum, but not hippocampus, may relate to the distinct origins of inhibitory neurons in these regions. In cortex, the majority of PVALB- and SST-containing inhibitory neurons are derived from the medial ganglionic eminence (MGE) (^71^, see^72^ for review). In hippocampus, although some PVALB- and SST-containing inhibitory neurons also arise from the MGE, approximately 40% of SST-containing inhibitory neurons are derived from the caudal ganglionic eminence (CGE)^73^. The dominant period of MGE neurogenesis (GD9.5-GD13.5) precedes that of CGE neurogenesis (GD12.5–16.5)^74,75^. It is possible that the time points we chose to elicit MIA may alter neurogenesis for each ganglionic eminence distinctly and somewhat spare the interneurons destined for the hippocampus.

We also present the first report of cortical and striatal *SSTR2* mRNA reductions in a rodent MIA model of schizophrenia, which aligns with the *SSTR2* mRNA reduction seen in the cortex of people with schizophrenia^26^. Reductions in both regions, however, are contrary to our hypothesis that postulated that the cortex is more susceptible to MIA than the striatum. Given that our previous behavioural study showed working memory impairments in GD19 polyI:C offspring, but not GD10 polyI:C offspring^50^, and multiple studies show a central role of somatostatinergic signalling in cognitive function^76–79^, our present findings suggest that the combined *SSTR2* mRNA deficits in cortex and striatum (found only at GD19), in combination with the *SST* mRNA deficits in cortex and striatum, may contribute to the working memory deficits apparent in late gestation MIA offspring. Qualitative observation of our data shows that *SST* mRNA deficits in cortex in polyI:C offspring seem to occur at both GD10 and GD19, whereas *SST* mRNA deficits in striatum in polyI:C offspring seem driven by MIA at GD10. This is possibly evidence of a timing-specific effect of MIA in striatum but not cortex; however, we did not detect a significant treatment × timing interaction effect or treatment × timing × subregion interaction effect that would permit us to investigate these further. Our data suggests that future studies are warranted with larger sample sizes may reveal significant effects of MIA timing on inhibitory markers in offspring.

Further, maternal IL-6 elevations are prolonged when polyI:C is administered in rodent dams during late gestation versus early gestation^80^, and maternal IL-6 levels in humans are associated with altered offspring neonatal functional connectivity and cognitive outcome^81–84^. Although we only measured maternal IL-6 at one time point, we also report a moderate negative relationship between maternal IL-6 and offspring *SST* gene expression in cortex and striatum. Overall, our data supports the notion that *SST* and *SSTR2* gene expression deficits may contribute to the working memory deficits apparent in late gestation MIA offspring.

Increased SST-positive interstitial white matter neurons (IWMN) and concurrent decreases in *SST* mRNA in the grey matter is reported in post-mortem frontal cortex from schizophrenia cases^24,85^. Recent studies show that both early gestation and late gestation polyI:C treatment increased the number of SST-positive IWMNs in regions that extend underneath the cingulate cortex in adult rat offspring^86^. Our detection of reduced *SST* mRNA in the grey matter suggests MIA during either the initial development of the ganglionic eminence^54^, or the time of neuronal tangential migration^55,56^ could alter the migration or survival of SST cortical interneurons. Interestingly, late gestation polyI:C offspring exhibit increased SST-positive IWMNs in more extensive regions of white matter compared to early gestation polyI:C offspring^86^. Given that SSTR2 is highly expressed on migrating neurons during early neurodevelopment in both rat and human^87^, our present findings may indicate a link between exaggerated IWMN pathology and SSTR2 deficits in late gestation polyI:C offspring. Indeed, reductions in *SST* mRNA and reductions in *SSTR2* mRNA are correlated in dlPFC in schizophrenia^26^.

Overall, our current and prior findings are consistent with our hypothesis and earlier findings that gestational inflammation contributes to inhibitory neurotransmission deficits. This supports the growing evidence of altered inhibitory indices in the cortex and striatum of adult polyI:C offspring and recapitulates some aspects of neuropathology present in schizophrenia. We present the first rodent MIA study of cortical and striatal deficits in *SST* and *SSTR2* gene expression that concurs with cortical *SST* and *SSTR2* mRNA deficits found in post-mortem schizophrenia studies. Our novel finding of *SST* and *SSTR2* mRNA reductions in striatum suggests that further examination of SST-related neurotransmission may be warranted in the striatum of people with schizophrenia. Although our data provide clues as to how inflammation, at different times during gestation, may contribute to schizophrenia-like pathologies, multiple gestational inflammatory insults and/or postnatal “second-hit” stressors are likely required to produce the large spectrum of symptoms and cognitive deficits that culminate in the diagnosis of schizophrenia. We postulate that somatostatinergic alterations could contribute to cognitive deficits previously reported in adult polyI:C offspring and in people with schizophrenia. Links between specific risk factors, such as maternal inflammation, and molecular alterations are critical to the development of mental illnesses, and we have identified SST and its receptor, SSTR2, as potential mediators of gestational inflammation-related risk for schizophrenia.

## Supplementary information

Supplementary figures and tables.

## Data Availability

The data analysed during the current study are available from the corresponding author on reasonable request.
