# Supplementary material for: Effect of Immune Activation during Early Gestation or Late Gestation on Inhibitory Markers in Adult Male Rats
Source: Sci Rep. 2020 Feb 6;10:1982. doi: 10.1038/s41598-020-58449-x (PMC7004984; doi:10.1038/s41598-020-58449-x)
Supplement: Supplementary file 1 — Supplementary figures and tables. [file 41598_2020_58449_MOESM1_ESM.docx]

#### Effect of Immune Activation during Early Gestation or Late Gestation on Inhibitory Markers in Adult Male Rats

Tasnim Rahman^a,b^, Cynthia Shannon Weickert^a,b,c^, Lauren Harms^d,e,f^, Crystal Meehan^d,e,f,g^, Ulrich Schall^e,f,h^ , Juanita Todd^d,e,f^ , Deborah M. Hodgson^d,e,f^, Patricia T. Michie^d,e,f^, and Tertia Purves-Tyson^a,b^.

^a^ School of Psychiatry, Faculty of Medicine, University of New South Wales, NSW, Australia.

^b^ Neuroscience Research Australia, NSW, Australia.

^c^ Department of Neuroscience and Physiology, State University of New York Upstate Medical University, Syracuse, NY, USA

^d^ School of Psychology, The University of Newcastle, NSW, Australia.

^e^ Priority Centre for Brain and Mental Health Research, The University of Newcastle, NSW, Australia.

^f^ Hunter Medical Research Institute, NSW, Australia.

^g^ Division of Psychology, School of Medicine, College of Health and Medicine, University of Tasmania, TAS, Australia.

^h^ School of Medicine and Public Health, The University of Newcastle, NSW, Australia

**Corresponding Author**:

Dr. Tertia Purves-Tyson

Neuroscience Research Australia, Randwick NSW 2031, Australia.

Tel: + 61 2 9 399 1751 Fax: + 61 2 9 399 1121

E-mail: t.purves-tyson@neura.edu.au

#### Supplementary materials

**
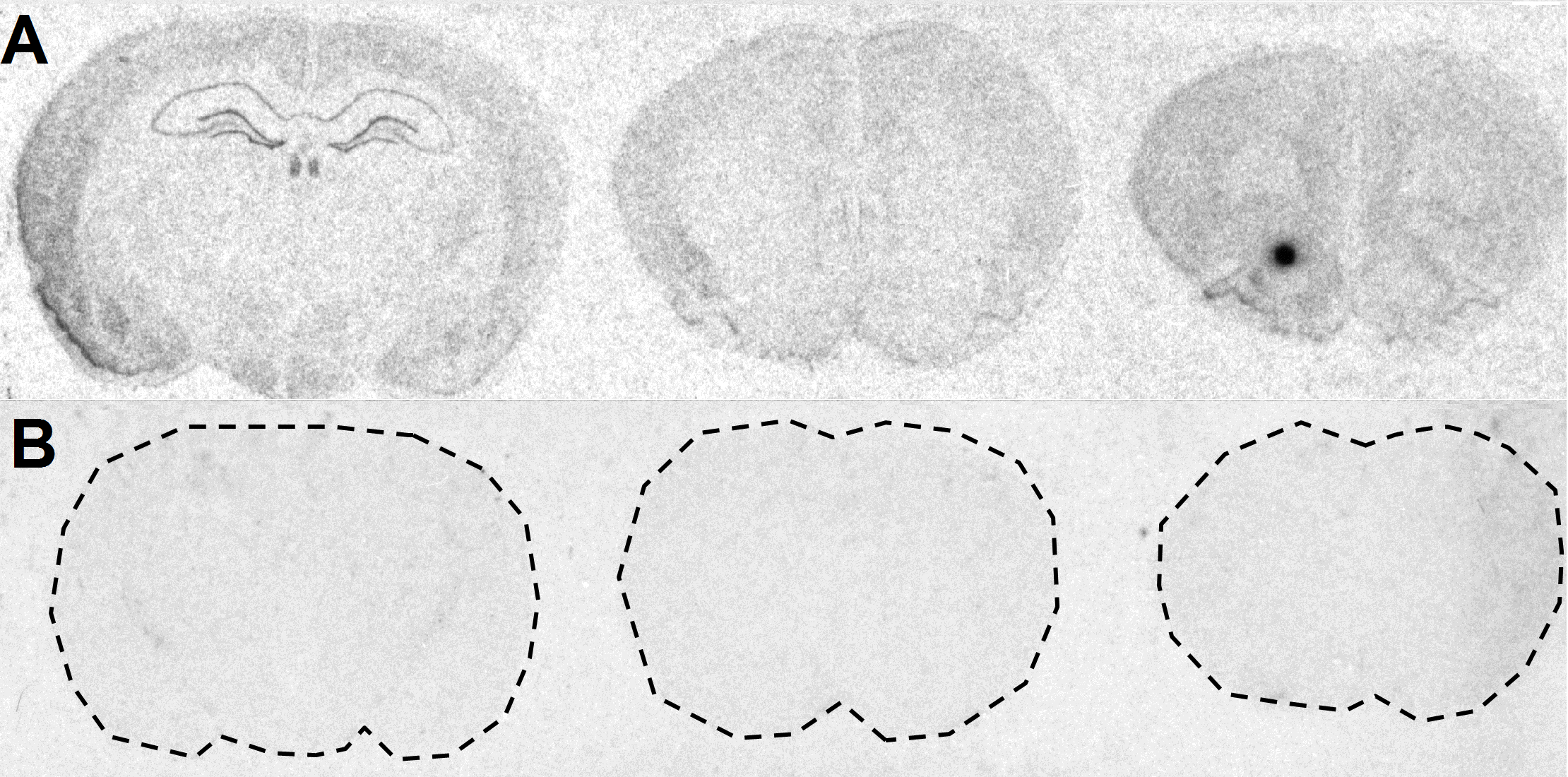
Supplementary Figure 1.** Autoradiographs of positive control (anti-sense strand, A) and negative control (sense strand, B) riboprobes for somatostatin receptor 2. The signal present in other brain regions (A) and the lack of signal in the coronal sections of the negative control (outlined in dashed black lines) (B) confirms that the signals from anti-sense riboprobes was specific and not due to background binding. Sections from left to right are from ~ -3.0 mm bregma, ~ 0.0 mm bregma, and ~ 3.0 mm bregma.


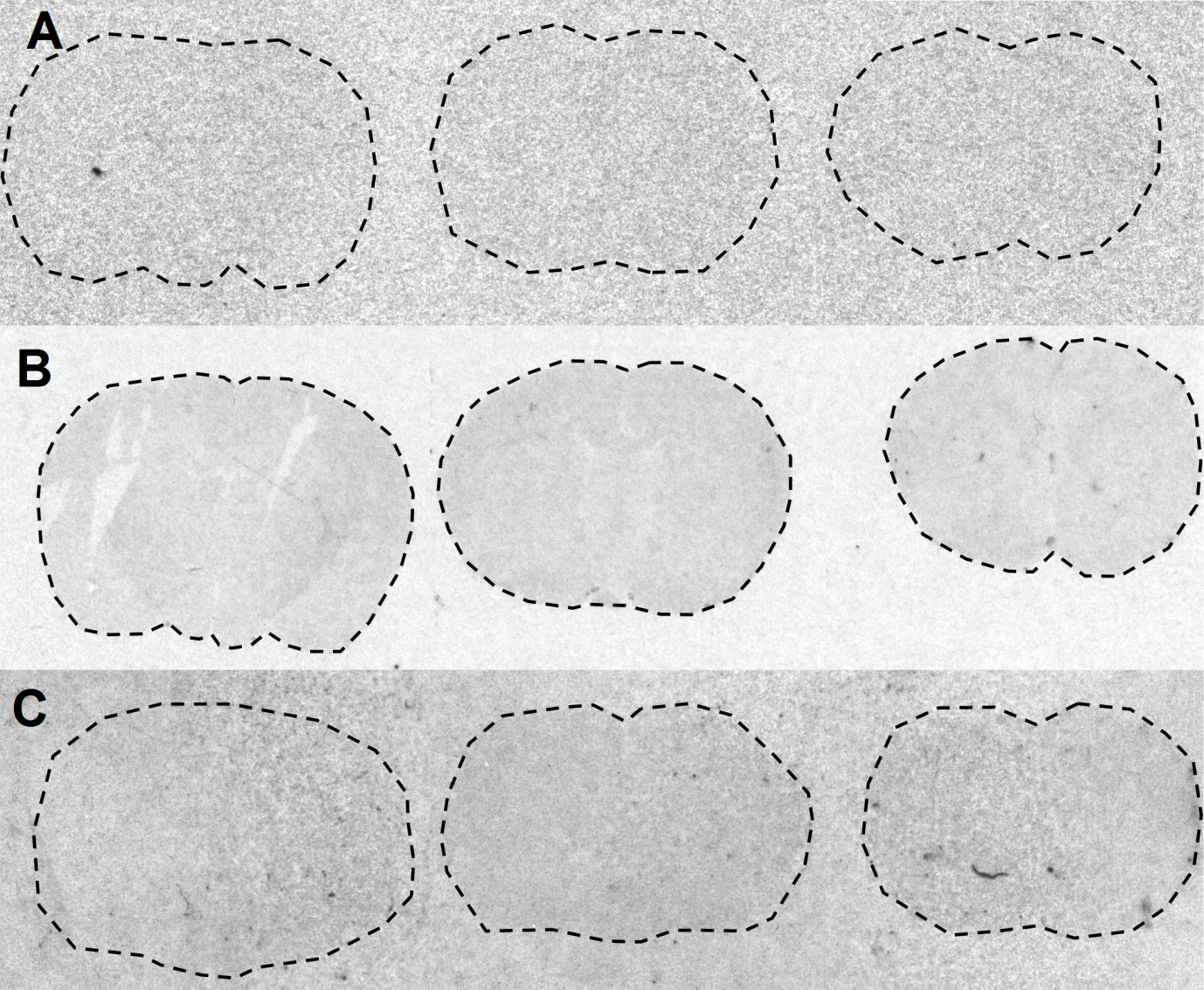


**Supplementary Figure 2.** Autoradiographs of negative control (sense strand) riboprobes for glutamate decarboxylase 1 (A), parvalbumin (B), and somatostatin (C). The lack of signal on the coronal sections (outlined in dashed black lines) confirms that the signals from anti-sense riboprobes for each target was specific and not due to background binding. Sections from left to right are from ~ -3.0 mm bregma, ~ 0.0 mm bregma, and ~ 3.0 mm bregma.


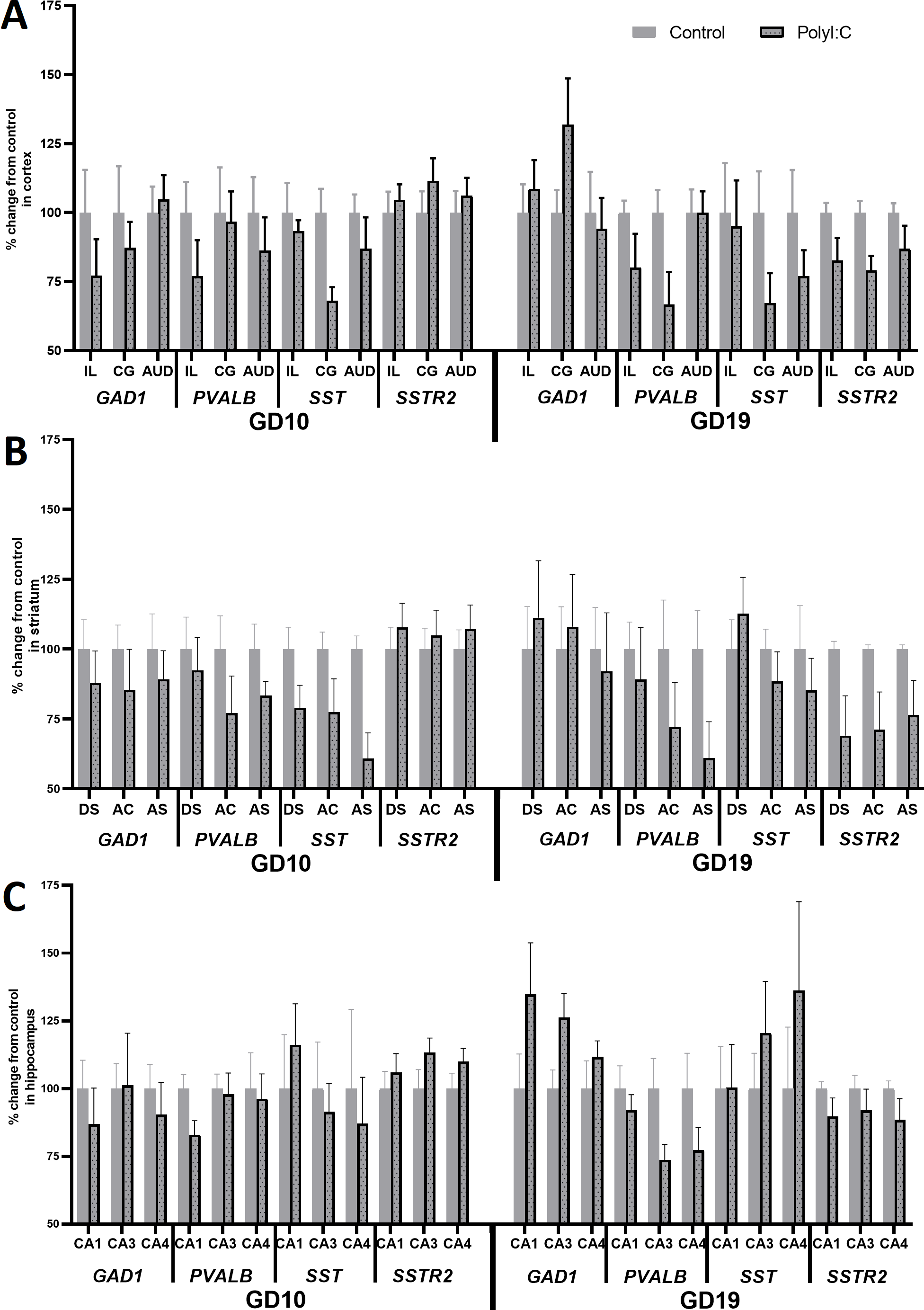


**Supplementary Figure 3.** Inhibitory neuron gene expression is not changed by MIA at a particular gestational timing in specific subregions of (A) cortex, (B) striatum, or (C) hippocampus in adult male offspring. Pregnant dams were treated with vehicle (Control, grey) or 4mg/kg (PolyI:C, dotted) during either early gestation (GD10, left) or late gestation (GD19, right). Glutamate decarboxylase 1 (GAD1) parvalbumin (PVALB), somatostatin (SST) and somatostatin receptor 2 (SSTR2) mRNAs were quantified in cortical (A), striatal (B), and hippocampal subregions in adult offspring. Treatment × subregion interactions were significant for SST in cortex and striatum (refer to Table 1 for statistics and Figure 4 for graphs). Treatment × timing × subregion interactions were not significant for any markers in any subregion (refer also to Table 1 for statistics). Data are mean ± SEM (n=6-8 rats per group). Infralimbic cortex (IL), cingulate cortex (CG), auditory cortex (AUD), dorsal striatum (DS), nucleus accumbens core (AC), nucleus accumbens shell (AS), cornu ammonis (CA).

**
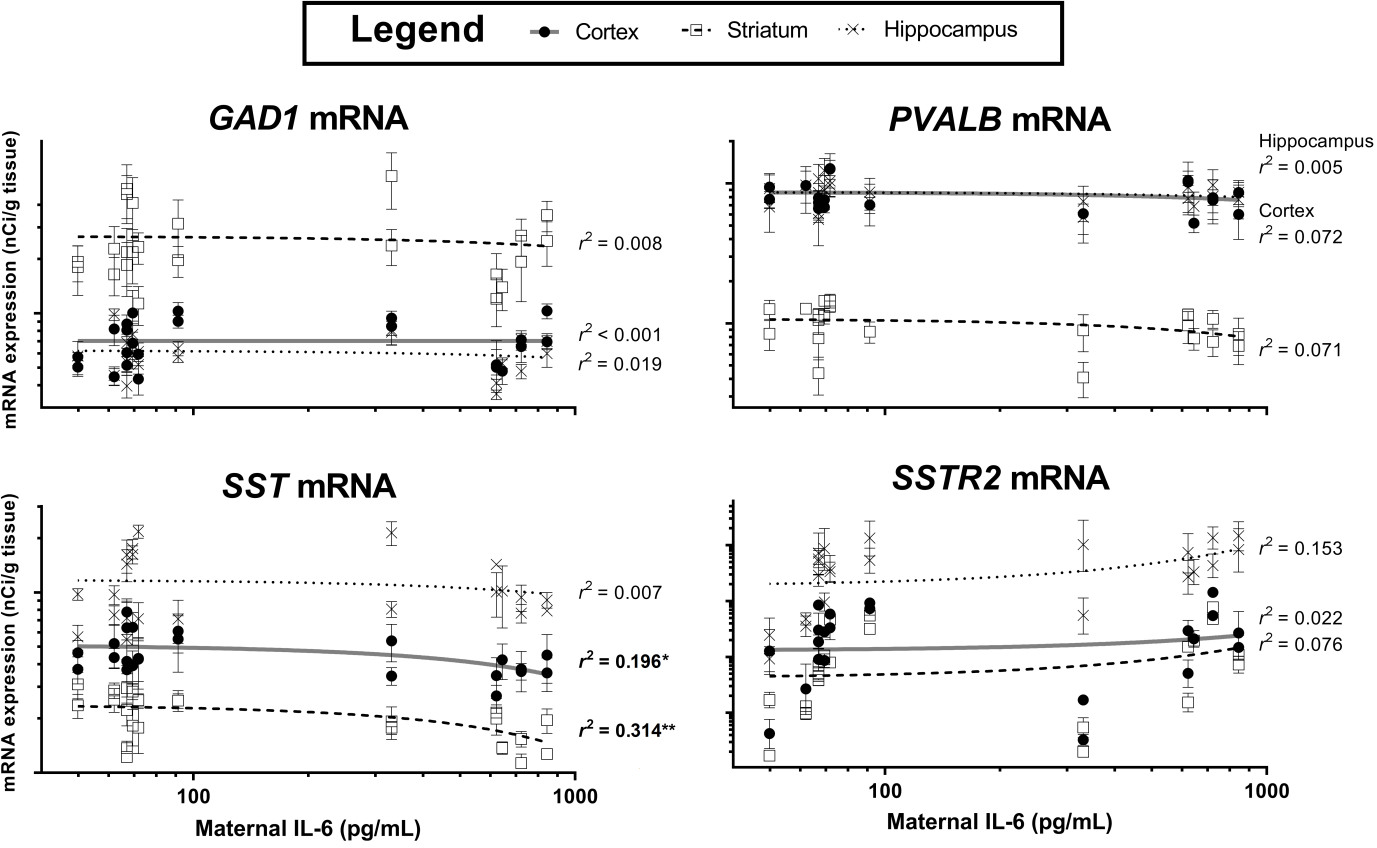
**

**Supplementary Figure 4.** Deming regression plots of comparisons between maternal IL-6 protein levels and gene expression in brain regions (cortex, striatum, hippocampus) of control and polyI:C offspring. Three subregions were used per region. Significant relationships were only found for *SST* mRNA in cortex and striatum brain regions. * *p* < 0.05

**Supplementary Table 1. Riboprobes for *in situ* hybridisation.**

| Target gene; and GenBank accession number | Riboprobe base pairs targeted; and % similarity to rat ortholog | Film exposure time (days) | Specific activity (cpm/µg) | |
| --- | --- | --- | --- | --- |
|  |  |  | Sense | Antisense |
| *GAD1*; NM_000817.2 | 1550-1839; 100% | 13 | 1.44×10^9^ | 1.28×10^9^ |
| *PVALB*; NM_002854.2 | 1-224; 84% | 16 | 1.38×10^9^ | 1.80×10^9^ |
| *SST*; NM_001048.3 | 207-446; 100% | 19 | 2.60×10^9^ | 2.23×10^9^ |
| *SSTR2*; NM_001050.2 | 690-982; 99% | 40 | 2.40×10^9^ | 1.80×10^9^ |

Supplementary Table 2. Subregional differences in gene expression. Cortex [infralimbic (IL), cingulate (Cg), and auditory (Aud)], striatum [dorsal striatum (DS), nucleus accumbens core (AC) and shell (AS)], and hippocampus [cornu ammonis area 1 (CA1), cornu ammonis area 3 (CA3), cornu ammonis area 4 (CA4),] from adult male control and polyI:C offspring. Regional differences of each mRNA are listed from least abundant to most abundant.

| mRNA and region | Statistics | Regional distribution |
| --- | --- | --- |
| *GAD1* Cortex | *F*(2,48) = 6.153, *p* < 0.01 | IL = Cg < Aud |
| *GAD1* Striatum | *F*(2,48) = 82.183, *p* < 0.001 | DS < AC < AS |
| *GAD1* Hippocampus | *F*(2,48) = 17.300, *p* < 0.001 | CA1 < CA3 < CA4 |
| *PVALB* Cortex | *F*(2,40) = 31.174, *p* < 0.001 | Cg < IL < Aud |
| *PVALB* Striatum | *F*(2,44) = 21.545, *p* < 0.001 | AC < AS < DS |
| *PVALB* Hippocampus | *F*(2,46) = 169.394, *p* < 0.001 | CA1 = CA3 < CA4 |
| *SST* Cortex | *F*(2,34) = 33.200, *p* < 0.001 | Aud < IL = Cg |
| *SST* Striatum | *F*(2,48) = 17.331, *p* < 0.001 | DS < AC < AS |
| *SST* Hippocampus | *F*(2,46) = 2.383, *p* > 0.05 | CA1 = CA3 = CA4 |
| *SSTR2* Cortex | *F*(2,46) = 7.171, *p* < 0.01 | IL = Aud < Cg |
| *SSTR2* Striatum | *F*(2,46) = 10.743, *p* < 0.001 | AS = AC < DS |
| *SSTR2* Hippocampus | *F*(2,48) = 185.701, *p* < 0.001 | CA1 = CA3 < CA4 |

Supplementary Table 3. Effect of MIA via polyI:C on inhibitory mRNAs and proteins in cortex and hippocampus in adult offspring. Methodological changes are listed for each study. Note that the administration of polyI:C (intravenously or intraperitoneally) is not listed and adds an additional level of variation. PL=prelimbic, IL=infralimbic, Cg=cingulate, mPFC=medial prefrontal cortex, PFC, prefrontal cortex.

| mRNA | Region | Mouse (M) or Rat (R) | GD | Subregion | Measure | Change? | Ref. |
| --- | --- | --- | --- | --- | --- | --- | --- |
| *GAD1* | Cortex | M  R  R  R  M | 9  14  14  15  17 | PL  IL  Cg  mPFC  PFC | Protein  mRNA  mRNA  Protein  mRNA | n.s  n.s  n.s  n.s.  **↓** | ^1^  ^2^  ^2^  ^3^  ^4,5^ |
| *GAD1* | Hippocampus | M  R  R  M | 17  14  15  17 |  | mRNA  mRNA  Cells  Protein | n.s  **↑**  **↓**  **↓** | ^6^  ^2^  ^3^  ^6^ |
| *PVALB* | Hippocampus | M  M  M  M  M  M  R  R | 9  9  9  17  12-15  15  15  15 |  | Cells  Cells  Cells  Cells  Cells  Cells  Cells  Cells | **↓**  n.s  n.s  n.s.  **↓**  **↓**  **↓**  **↓** | ^7^  ^8^  ^9^  ^9^  ^10^  ^11^  ^3^  ^12^ |

1 Canetta, S. *et al.* Maternal immune activation leads to selective functional deficits in offspring parvalbumin interneurons. *Molecular Psychiatry*, doi:10.1038/mp.2015.222 (2016).

2 Cassella, S. N. *et al.* Maternal immune activation alters glutamic acid decarboxylase-67 expression in the brains of adult rat offspring. *Schizophrenia Research* **171**, 195-199, doi:10.1016/j.schres.2016.01.041 (2016).

3 Dickerson, D. D. *et al.* Association of aberrant neural synchrony and altered GAD67 expression following exposure to maternal immune activation, a risk factor for schizophrenia. *Transl Psychiatry* **4**, e418-, doi:10.1038/tp.2014.64 (2014).

4 Labouesse, M. A., Dong, E., Grayson, D. R., Guidotti, A. & Meyer, U. Maternal immune activation induces GAD1 and GAD2 promoter remodeling in the offspring prefrontal cortex. *Epigenetics* **10**, 1143-1155, doi:10.1080/15592294.2015.1114202 (2015).

5 Richetto, J., Calabrese, F., Riva, M. A. & Meyer, U. Prenatal immune activation induces maturation-dependent alterations in the prefrontal GABAergic transcriptome. *Schizophrenia bulletin* **40**, 351-361, doi:10.1093/schbul/sbs195 (2014).

6 Luoni, A., Richetto, J., Longo, L. & Riva, M. A. Chronic lurasidone treatment normalizes GABAergic marker alterations in the dorsal hippocampus of mice exposed to prenatal immune activation. *Eur Neuropsychopharmacol* **27**, 170-179, doi:10.1016/j.euroneuro.2016.12.001 (2017).

7 Ducharme, G., Lowe, G. C., Goutagny, R. & Williams, S. Early alterations in hippocampal circuitry and theta rhythm generation in a mouse model of prenatal infection: implications for schizophrenia. *PLoS One* **7**, e29754, doi:10.1371/journal.pone.0029754 (2012).

8 Giovanoli, S., Weber, L. & Meyer, U. Single and combined effects of prenatal immune activation and peripubertal stress on parvalbumin and reelin expression in the hippocampal formation. *Brain Behav Immun* **40**, 48-54, doi:10.1016/j.bbi.2014.04.005 (2014).

9 Meyer, U., Nyffeler, M., Yee, B. K., Knuesel, I. & Feldon, J. Adult brain and behavioral pathological markers of prenatal immune challenge during early/middle and late fetal development in mice. *Brain, behavior, and immunity* **22**, 469-486, doi:10.1016/j.bbi.2007.09.012 (2008).

10 Han, M. *et al.* Intake of 7,8-Dihydroxyflavone During Juvenile and Adolescent Stages Prevents Onset of Psychosis in Adult Offspring After Maternal Immune Activation. *Scientific reports* **6**, 36087, doi:10.1038/srep36087 (2016).

11 Zhang, Z. & van Praag, H. Maternal immune activation differentially impacts mature and adult-born hippocampal neurons in male mice. *Brain Behav Immun* **45**, 60-70, doi:10.1016/j.bbi.2014.10.010 (2015).

12 Piontkewitz, Y. *et al.* Effects of risperidone treatment in adolescence on hippocampal neurogenesis, parvalbumin expression, and vascularization following prenatal immune activation in rats. *Brain, Behavior, and Immunity* **26**, 353-363, doi:10.1016/j.bbi.2011.11.004 (2012).
